# Construction and validation of a prediction model for the risk of citrate accumulation in patients with hepatic insufficiency receiving continuous renal replacement therapy with citrate anticoagulation

**DOI:** 10.1186/s12882-024-03462-9

**Published:** 2024-01-22

**Authors:** Quxia Hong, Siyu Chen, Yongchun He, Jianghua Chen, Ping Zhang

**Affiliations:** 1 Kidney Disease Center, First Affiliated Hospital, College of Medicine, Zhejiang University, Hangzhou, Zhejiang China; 2 Key Laboratory of Kidney Disease Prevention and Control Technology, Hangzhou, Zhejiang China; 3 Institute of Nephrology, Zhejiang University, Hangzhou, Zhejiang China; 4 Zhejiang Clinical Research Center of Kidney and Urinary System Disease, Hangzhou, Zhejiang China; 5 Department of Nephrology, Tiantai People’s Hospital, Taizhou, Zhejiang, China

**Keywords:** Citrate accumulation, Hepatic insufficiency, CRRT, Regional citrate anticoagulant

## Abstract

**Background:**

To construct and validate a prediction model of the risk of citrate accumulation in patients with hepatic dysfunction receiving continuous renal replacement therapy with regional citrate anticoagulation (RCA-CRRT), which reduces the risk of citrate accumulation.

**Methods:**

All patients who received RCA-CRRT from 2021 to 2022 and were hospitalized in the First Affiliated Hospital of Zhejiang University were considered for study participation. Logistic regression analysis was used to identify the risk factors for citrate accumulation, based on which a nomogram model was constructed and validated in the validation group.

**Results:**

Six factors were finally identified, from which a nomogram was created to predict the risk of citrate accumulation. The area under the curve of the prediction model was 0.814 in the training group and 0.819 in the validation group, and the model showed acceptable agreement between the actual and predicted probabilities. Decision curve analysis also demonstrated that the model was clinically useful.

**Conclusions:**

The model constructed from six factors reliably predicted the risk of citrate accumulation in patients with hepatic insufficiency who received RCA-CRRT.

## Introduction

Safe and effective anticoagulation ensures the success of continuous renal replacement therapy (CRRT), which is commonly used for critically ill patients. Systemic heparin anticoagulation and regional citrate anticoagulation (RCA) are the two main anticoagulation strategies used today, and previous studies have shown that RCA can improve the filter life span and reduce the risk of bleeding significantly when compared to the former [1, 2]. The 2012 Kidney Disease: Improving Global Outcomes (KDIGO) guidelines also regard citrate as the preferred anticoagulant for patients at increased bleeding risk without contraindications to citrate [3]. Citrate metabolism takes place in organs with high amounts of mitochondria, such as the liver, kidneys and muscles [4, 5]. Although the filter removes some of the citrate, a certain amount of citrate can still run into the systemic circulation. As some related clinical studies have reported, citrate clearance is significantly impaired in critically ill patients with decompensated hepatic failure,which is why citrate accumulation, defined as citrate-induced toxicity during CRRT, is prone to occur in hepatic failure patients [6–8]. Therefore, severe liver failure is regarded as a contraindication to citrate anticoagulation [3]. Considering that the accumulation of citrate could lead to electrolyte disorders and acid–base imbalance and even affect the mortality of patients [9, 10], although RCA-CRRT might be safe and effective in hepatic failure patients when electrolytes and blood gases are monitored closely [11, 12], avoiding citrate accumulation is still the main concern when citrate is used. In addition, citrate accumulation is thought to be the result of multiple factors, not only hepatic failure leading to the impaired metabolism of citrate [13]. The probability of citrate accumulation in patients with hepatic failure undergoing RCA-CRRT should be estimated and predicted by a model to develop an improved method for CRRT. The objective of this study was to explore a prediction model that is visualized via nomograms by analyzing the independent risk factors for citrate accumulation in patients with hepatic insufficiency, which is important for formulating appropriate initial CRRT prescriptions in clinical practice.

## Materials and methods

### Study samples

All patients treated with RCA-CRRT from January 2021 to November 2022 which were hospitalized in the intensive care unit of our institution, the First Affiliated Hospital of Zhejiang University, were included in the study. The inclusion criteria were as follows: (1) a prescription for RCA-CRRT for renal replacement therapy; (2) The patients with hepatic insufficiency (impaired liver function and abnormal imaging findings by any causes of liver cirrhosis and liver diseases). Patients with varying degrees of hepatic insufficiency which were defined as total bilirubin (TB) was greater than 2 mg/dL or Child-pughB、Child-pughC [14–16]. In addition, patients who met the following criteria were excluded from the analysis: (1) age < 18 years old; (2) use of RCA for less than 24 hours due to surgery or examination; and (3) treatment with other anticoagulants in combination with RCA initially. The events per variable (EPV) method was used to calculate the sample size. Based on previous research, 10 risk factors were predicted. The EPV value was set as 5 to ensure the stability of the results. It could be concluded that at least 50 positive samples were needed for the study, and the positive incidence rate was approximately 33% according to relevant studies. Therefore, the sample size for modeling should not be less than 152 cases. To evaluate the generalization of the prediction model, the samples were divided into training and validation groups using time series. The training group included patients from January 2021 to July 2022, and the validation group included patients from August 2022 to November 2022.

### Study variables

General demographic characteristics, such as age, sex, weight, body mass index and blood pressure, including systolic, diastolic and mean arterial pressure (MAP), were collected at the beginning of RCA-CRRT. The hospitalization records were reviewed to determine the primary diseases causing hepatic insufficiency, indications for treatment with CRRT and therapeutic parameters for CRRT, including the dialysate flow rate, replacement fluid flow rate, blood flow rate and citrate concentration in peripheral blood. In addition, the Acute Physiology And Chronic Health Evaluation (APACHE) II score, Sequential Organ Failure Assessment (SOFA) score, Model for End-Stage Liver Disease (MELD) score, and Child–Pugh score of patients and the peak norepinephrine dose while receiving CRRT were also recorded.

The following parameters were also measured: white blood cell count, hemoglobin, platelet (PLT) count, C-reactive protein (CRP), albumin (Alb), alanine aminotransferase (ALT), aspartate aminotransferase (AST), total bilirubin (Tbil), lactate dehydrogenase (LDH), activated partial thromboplastin time (APTT), prothrombin time (PT), international normalized ratio (INR), serum creatinine (SCr), blood urea nitrogen (BUN), serum potassium, total calcium concentration (tCa), and blood gases before and after CRRT treatment, including pH, partial pressure of oxygen (PO2), partial pressure of carbon dioxide (PCO2), base excess, lactate, bicarbonate concentration and ionized calcium concentration(iCa). Among them, blood gases were monitored at 4-hour intervals, and the total serum calcium concentration was monitored at 24 hours.

### Definitions

A total-to-ionized calcium (T/iCa) ratio of ≥2.5 was considered to indicate citrate accumulation [17, 18], and patients were divided into the accumulation group and the control group.

### CRRT mode and parameters

A temporary hemodialysis tube in the femoral or internal jugular vein was used in all patients treated with the modality of continuous venovenous hemofiltration or continuous venovenous hemodiafiltration. The blood flow rate was set as 160 ml/min, the replacement fluid rate as 1000–2000 ml/h and the dialysate flow rate as 1000–3000 ml/h initially. For anticoagulation, 4% trisodium citrate solution was infused into the arterial line of the extracorporeal circuit, and the initial dose was set according to the patient’s condition, such as hepatic function and blood lactate levels. To maintain a fixed dose of citric acid during the whole treatment unless citrate accumulation, so we don’t need to monitor the circuit blood citrate concentration during the whole treatment. Replacement fluid containing 1.5 mmol/L calcium was applied in both the dialysate and replacement fluid. The calcium gluconate solution (10%) was continuously infused and adjusted by the calcium ion level when monitored every 4 hours to be maintained at 0.9–1.1 mmol/L. At the same time, sodium bicarbonate solution (5%) was added and adjusted according to the acid–base status of the patient.

### Statistical analysis

Normally distributed variables were expressed as the mean ± SD, and comparisons between groups were performed by t test. Skewed data was expressed as the median (interquartile range), and the Mann–Whitney U nonparametric test was used for intergroup comparisons. Categorical variables were expressed as frequencies and percentages, and differences between groups were analyzed by chi-square tests. The variance inflation factor (VIF) was used to evaluate screening factors for multicollinearity. Variables with *P* < 0.1 assessed by univariable logistic regression analysis were entered into the multivariable logistic regression analysis to identify risk factors for citrate accumulation. A nomogram model including risk factors to predict the probability of citrate accumulation was constructed, the performance of which was evaluated by discrimination and calibration. The area under the receiver operating characteristic (ROC) curve (AUC) was used to reflect the discriminative ability of the model, and calibration curves were generated to reflect the calibration of the model. We also used decision curve analysis (DCA) to evaluate the clinical usefulness of the model. Our model was then verified in another group of patients. *P* < 0.05 was considered to indicate a statistically significant difference. Statistical analysis was performed using SPSS 25.0 and R 4.2.2.

## Results

### Demographic and clinical characteristics

**Fig. 1:**
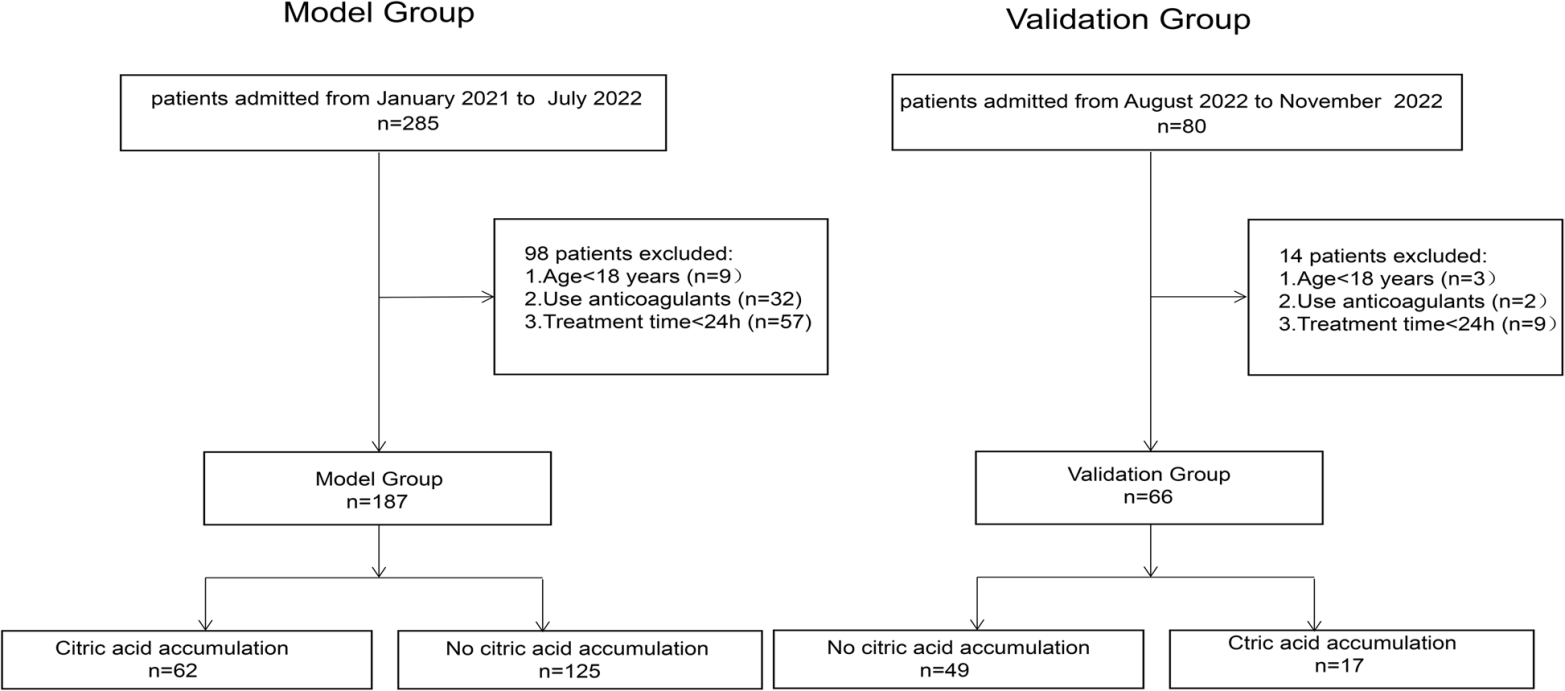
Flowchart of the study participants

A total of 253 patients were enrolled, including 187 patients in the training group and 66 patients in the validation group. There were 62 patients (33.2%) in the training group and 17 patients (25.8%) in the validation group with citrate accumulation. A flow diagram showing the research design is shown in Fig. 1.

Among 253 patients, the circuit blood citrate concentration was 1.5 mmol/L in 23 patients, 1.6–2.0 mmol/L in 48 patients, 2.1–2.9 mmol/L in 42 patients and ≥ 3.0 mmol/L in 140 patients. The life span of the filter and pipeline in each group was 60 h (56,69), 60 h (48,69), 48 h (36,72), and 71 h (56,72). A total of 57 patients who discontinued therapy midway due to surgery or examination were not included in the final analysis of filter life span. More characteristics of the patients are presented in Table 1.

**Table 1:** Life span of the filter and pipeline by peripheral blood citrate concentration

Peripheral blood citrate concentration(mmol/L)	All patients(*n* = 253)	Life span of filter and pipeline (h)	Discontinued midway due to surgery or examination, n(%)
=1.5	23	60(56,69)	9(15.8
1.6–2.0	48	60(48,69)	8(14)
2.1–2.9	42	48(36,72)	11(19.3)
≥3.0	140	71(56,72)	29(50.9)

### Risk factors predicting citrate accumulation

Patients in the training group were divided into two groups according to whether citrate accumulation occurred. There were significant differences in age, sex, MAP, norepinephrine dosage, INR, Tbil, (tCa), PO2, PCO2, peripheral blood citrate concentration, APACHE II score, SOFA score and Child–Pugh score between the two groups, as described in Table 2.

**Table 2:** Clinical characteristics of the training group

Characteristics	All patientss (*n* = 187)	Control group(*n* = 125)	Accumulation group (*n* = 62)	Z/χ2	*P*-value
Age (years)	56(46, 66)	54(43,63)	62(53.25,70)	−3.06	0.002
Male【n(%)】	128(68.4)	91(72.8)	37(59.7)	2.542	0.069
Hepatic dysfunction classification【n(%)】				4.793	0.309
acute hepatic failure	52(27.8)	39(31.2)	13(21)		
subacute hepatic failure	3(1.6)	1(0.8)	2(3.2)		
acute-on-chronic hepatic failure	37(19.8)	21(16.8)	16(25.8)		
chronic hepatic failure	34(18.2)	23(18.4)	11(17.7)		
hepatic dysfunction	61(32.6)	41(32.8)	20(32.3)		
Indications for CRRT 【n(%)】				0.586	0.746
CKD	11(5.9)	8(6.4)	3(4.7)		
AKI	129(69)	84(67.2)	45(72.6)		
Others	47(25.1)	33(26.4)	14(21.9)		
APACHE II scores	23.6 ± 6.18	22.53 ± 5.85	25.73 ± 5.80	−3.415	0.001
MELD scores	22.8(20.45,25.60)	26.5(21,31)	30(24,34)	−3.297	0.001
SOFA	12(10,14)	12(10,14)	12.5(10,15)	−0.892	0.375
Child-pugh	9(8,11)	9(7.5,10.5)	10(8,11)	−1.701	0.089
BMI (kg/m^2)	22.7(20.3,25.60)	22.85(20.48,25.6)	22.4(19.6,25.6)	−0.253	0.801
Weight (kg)	62.75(55,70)	63(55,71)	60(50,70)	−1.448	0.148
Map (mmHg)	81(74,89)	81(75,89)	78(71,86.25)	−2.342	0.019
Norepinephrine (ug/kg/min)	0.02(0, 0.16)	0.00(0,0.11)	0.08(0, 0.2)	−2.299	0.016
Alb (g/L)	33.1(29.3,36.6)	33.1(29.4,37.6)	32.9(29.3,35.50)	−0.818	0.413
AST (U/L)	127(53, 438)	143(54.5548.3)	107(44.5325.5)	−1.108	0.268
ALT (U/L)	102(33, 387)	122(35,459)	55(29.5231.8)	−1.623	0.105
LDH (U/L)	399(284,758)	395(273,736)	403(296, 857)	−0.353	0.724
Tbil (umol/L)	130(57,257)	126.4(55.6239.4)	138.3(60.9320.8)	−0.796	0.426
CRP (mg/L)	53.26(20.15,101.55)	53.7(23.6101.9)	43.8(16.9100.9)	−1.020	0.308
WBC(*10^9/L)	9.65(6.5,15.24)	9.85(6.49,15.67)	9.34(6.57,13.92)	−0.065	0.949
PLT(*10^9/L)	46(29,106)	52(30,121)	40(27.25,83.5)	−1.894	0.058
Hb(g/L)	73(62,93)	73(60,96)	71.5(63,92.25)	−0.675	0.5
PT(s)	18.1(14.6,24.3)	17.4(14.6,23.1)	19.8(15.4,27)	−1.765	0.078
INR	1.64(1.28,2.22)	1.54(1.26,2.12)	1.93(1.32,2.62)	−2.292	0.022
APTT(s)	44.1(33.2,60.1)	43.1(33.2,67.3)	46.4(33.2,67.3)	−1.280	0.201
serum potassium (mmol/L)	4.32(4,4.67)	4.34(3.99,4.69)	4.32(4.06,4.62)	−0.323	0.747
tCa (mmol/L)	2.25(2.17,2.25)	2.23(2.16, 2.36)	2.27(2.20,2.45)	−2.173	0.03
Scr (umol/L)	168(87,255)	158(78, 254)	175(94.5267)	−1.423	0.155
BUN (mmol/L)	14.6(7.9,14.6)	13.5(7.59,20.74)	15.11(7.68,23.55)	−1.082	0.279
pH	7.43(7.38,7.47)	7.43(7.38,7.46)	7.45(7.39,7.48)	−1.735	0.083
PO2 (mmHg)	106(89,132)	113(91,139)	99.5(80,118)	−2.569	0.01
PCO2 (mmHg)	34.8(31.6,39.2)	35(32,40.03)	33.9(30.65,38.55)	−1.613	0.107
Bicarbonate concentration (mmol/L)	23.16 ± 3.8	23 ± 4.05	23.2 ± 3.3	−0.079	0.937
BE (mmol/L)	−1.15 ± 4.13	−1.3 ± 4.38	−0.93 ± 3.65	− 0.506	0.613
Lactate (mmol/L)	1.9(1.3,3.1)	1.8(1.2,2.8)	2.2(1.3,3.9)	−1.515	0.13
iCa (mmol/L)	1.12(1.04,1.18)	1.12(1.04,1.17)	1.11(1.03,1.18)	−0.154	0.879
Dialysate flow rate (ml/h)	1000(1000,2000)	1000(1000,2000)	1000(1000,2000)	−2.089	0.037
replacement flow rate (ml/h)	1000(1000,1000)	1000(1000,1000)	1000(1000,1000)	−0.329	0.742
CRRT dosage (ml/h/kg)	38(31,50)	40(32,50)	37(30,48)	−0.897	0.370
Citrate concentration (mmol/L)	3(2,3)	2.5(2,3)	3(2.5,3)	−2.792	0.005

*CRRT* continuous renal replacement therapy, *AKI* acute kidney injury, *CK* chronic kidney disease, *APACHE II* Acute Physiology and Chronic Health Evaluation, *MELD* Model for End-Stage Liver Disease, *SOFA* Sequential Organ Failure Assessment, *BMI* body mass index, *MAP* mean arterial pressure, *Alb* albumin: *ALT* alanine aminotransferase, *AST* aspartate aminotransferase, *Tbil* total bilirubin, *LDH* lactate dehydrogenase, *WBC* white blood cell count, *Hb* hemoglobin, *PLT* platelet count, *CRP* C-reactive protein, *APTT* activated partial thromboplastin time, *PT* prothrombin time, *INR* international normalized ratio, *Sc*r serum creatinine, *BUN* blood urea nitrogen, *tCa* total calcium concentration, *O2* partial pressure of oxygen, *PCO2* partial pressure of carbon dioxide, *BE* Base excess, *iCa* ionized calcium concentration

The APACHE II, MELD and Child–Pugh scores were not included in the model construction to reduce the impact of repeated indicators. All of the above factors with *P* < 0.5 were divided into two or multiple categories, including age according to the “Analysis on the current situation of population aging “ [19], Weight, AST, ALT, CRP, PLT count, PT, APTT, Alb, serum calcium, SCr, BUN, PH, PCO2 and dialysate flow rate as the median or mean, Tbil and INR according to the severity of hepatic failure [20], PO2 and MAP according to the lower limit of normal, and norepinephrine dosage and lactic acid according to relevant references [21, 22].

The VIF was used to evaluate screened variables for multicollinearity, and all variables except PT(VIF > 10) were finally included in our model. Variables with *P* < 0.1 screened by univariable logistic regression were included in the multivariate logistic regression. Six variables (sex, INR, norepinephrine dosage, PO2, dialysate flow rate and peripheral blood citrate concentration) were finally identified as risk factors for citrate accumulation, as shown in Table 3.

**Table 3:** Univariate and multivariate regression for prognostic factors

Variables	Univariate regression			*P*-value	Multivariate regression			*P*-value
	0R(95%CI)	estimates	Wald		0R(95%CI)	estimates	Wald	
Female	1.808(0.951–3.438)	0.592	3.267	0.071	2.405(1.041–5.556)	0.878	4.223	0.040
Age ≥ 65 years	2.502(1.297–4.825)	0.917	7.492	0.006				
Weight <63 (kg)	1.124(0.541–2.337)	0.117	0.098	0.755				
Map <70 mmHg	2.497(0.921–4.807)	0.744	3.110	0.078				
norepinephrine≥0.1μg/kg/min	2.004(1.311–4.756)	0.915	7.753	0.005	3.005(1.326–6.809)	1.100	6.949	0.008
AST ≥ 127(U/L)	0.76(0.413–1.4)	−0.274	0.774	0.379				
ALT≥102(U/L)	0.566(0.305–1.05)	−0.568	3.255	0.071				
Tbil <171umol/L			2.159	0.34				
Tbil 171-342umol/L	0.895(0.432–1.851)	−0.111	0.09	0.764				
Tbil >342umol/L	1.744(0.755–4.030)	0.556	1.696	0.193				
PT ≥ 18 s	1.766(0.952–3.274)	0.568	3.255	0.071				
APPT≥44 s	1.45(0.786–2.676)	0.372	1.413	0.234				
INR < 1.9			8.478	0.014			20.321	< 0.001
INR 1.9–2.6	2.029(0.974–4.228)	0.708	3.57	0.059	5.770(2.030–16.400)	1.753	10.813	0.001
INR > 2.6	3.142(1.354–7.285)	1.144	7.104	0.008	14.891(4.237–52.336)	2.701	17.737	< 0.001
PLT <46*10^9/L	1.599(0.865–2.958)	0.47	2.242	0.134				
CRP ≥ 53.26(mg/L)	0.824(0.447–1.517)	−0.194	0.387	0.534				
Alb < 33(g/L)	1.016(0.553–1.868)	0.016	0.003	0.959				
tCa ≥ 2.3 mmol/L	1.490(0.791–2.806)	0.399	1.524	0.217				
Scr ≥ 168umol/L	1.542(0.838–2.855)	0.436	1.942	0.163				
BUN≥15 mmol/L	1.255(0.681–2.311)	0.227	0.529	0.467				
PH ≥ 7.43	1.821(0.983–3.371)	0.599	3.631	0.057				
PO2 <80 mmHg	3.005(1.308–6.905)	1.1	6.722	0.01	3.971(1.341–11.757)	1.373	6.199	0.013
Lactate≥4(mmol/L)	1.203(0.533–2.716)	0.185	0.198	0.656				
PCO2 ≥ 35 mmHg	0.732(0.395–1.357)	− 0.312	0.982	0.322				
Citrate concentration = 1.5 mmol/L			7.842	0.049			13.060	0.005
Citrate concentration 1.6-2 mmol/L	1.615(0.368–7.096)	0.48	0.403	0.346	5.715(0.813–40.151)	1.743	3.071	0.080
Citrate concentration 2.1–2.9 mmol/L	3(0.736–12.227)	1.099	2.349	0.088	14.892(2.167–102.35)	2.701	7.541	0.006
Citrate concentration ≥ 3 mmol/L	4.21(1.162–15.256)	1.438	4.79	0.025	19.183(3.209–114.678)	2.954	10.484	0.001
Dialysate flow rate ≤ 1000 ml/h	1.861(0.989–3.502)	0.547	3.708	0.054	3.085(1.319–7.212)	1.126	6.758	0.009
constant					0.003	−5.978	24.649	< 0.001

*MAP* mean arterial pressure, *Alb* albumin, *ALT* alanine aminotransferase, *AST* aspartate aminotransferase, *Tbil* total bilirubin, *PLT* platelet count, *CRP* C-reactive protein, *PT* prothrombin time, *INR* international normalized ratio, *Scr* serum creatinine, *BUN* blood urea nitrogen, *tCa* total calcium concentration, *PO2* partial pressure of oxygen, *PCO2* partial pressure of carbon dioxide

### Nomogram and model performance

**Fig. 2:**
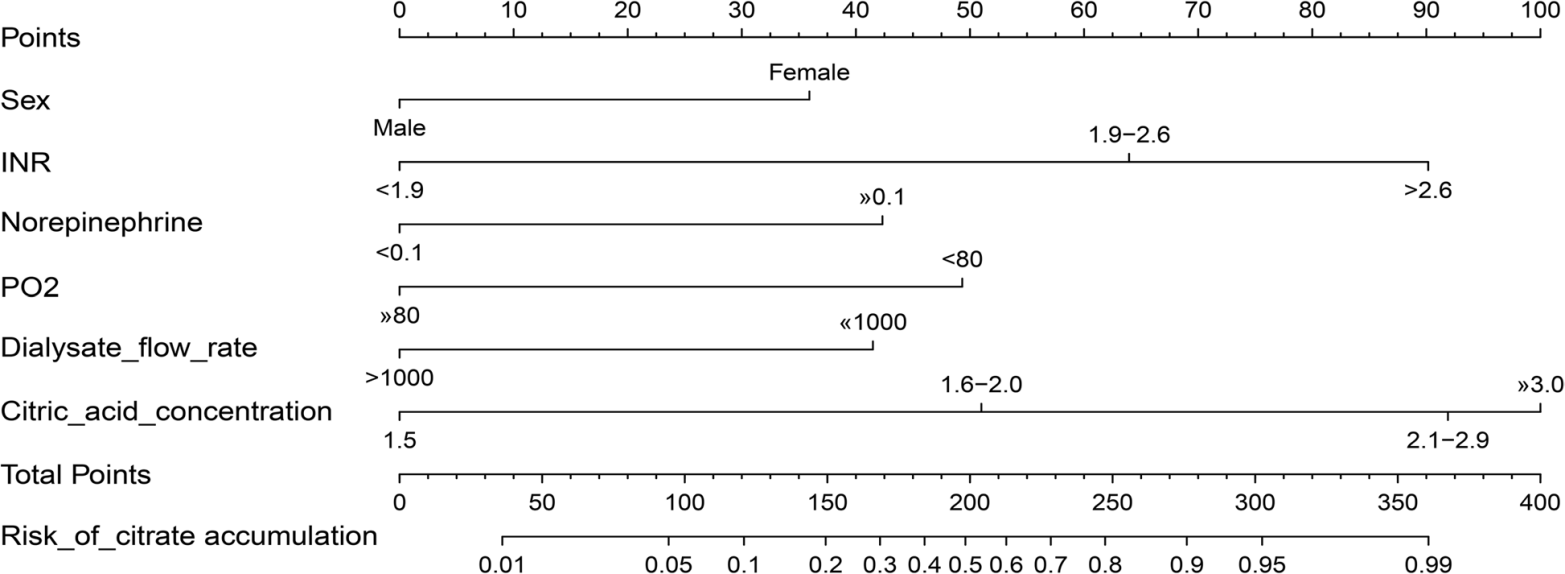
The nomogram for predicting the risk of citrate accumulation in patients underwent RCA-CRRT. Each level of predictor indicates a certain score. A total score was generated by a summary of the score of each predictor. The total score corresponds to hyperkalemia probability. *Tbil* total bilirubin: *INR* international normalized ratio: *PO2* partial pressure of oxygen

In accordance with the multivariable logistic regression analysis, a nomogram was created to predict citrate accumulation in patients who underwent RCA-CRRT, including 6 significant risk factors, as shown in Fig. 2.

**Fig. 3:**
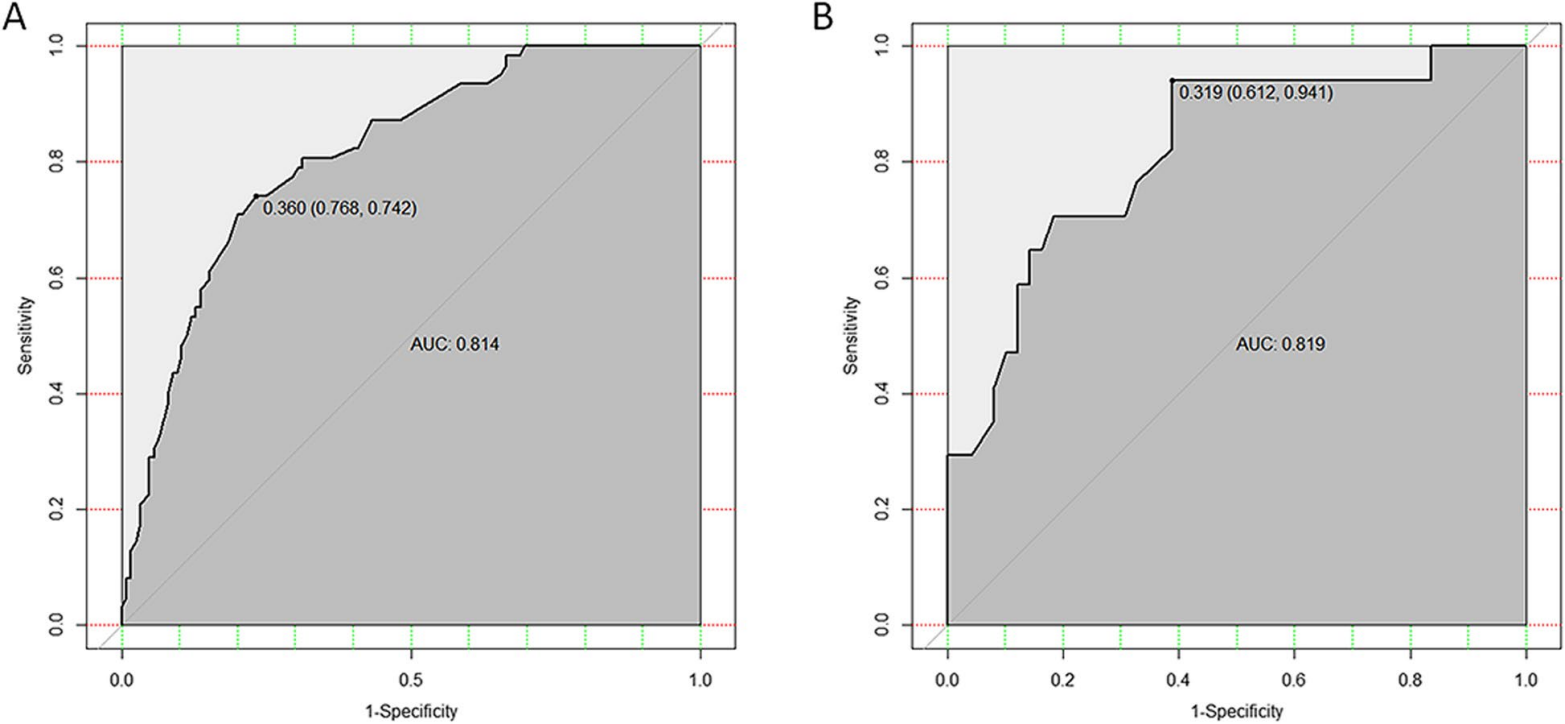
ROC curve and AUC of the predictive model. A The ROC in the training group. B The ROC in the validation group. ROC: receiver operating characteristic; AUC: area under the curve

**Fig. 4:**
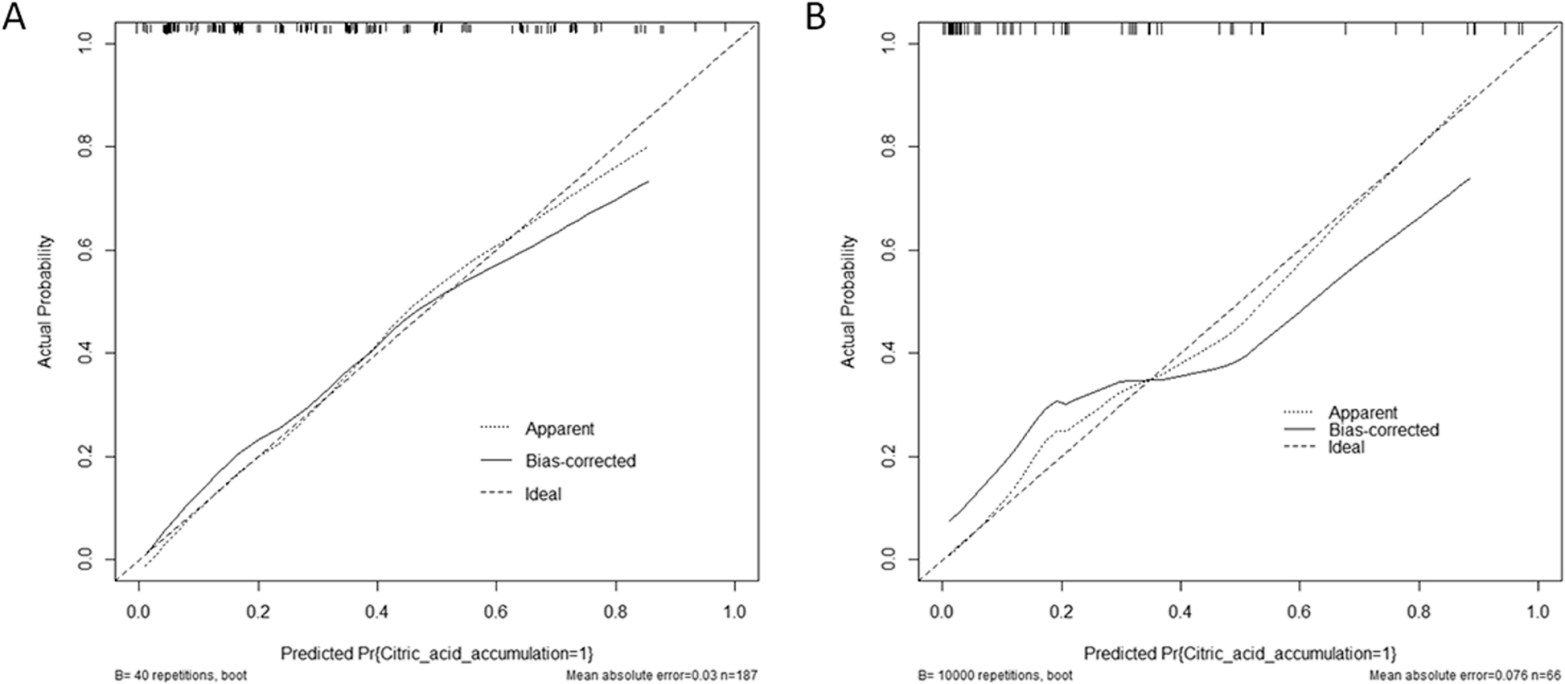
Calibration plots of the predictive model. **a** Calibration plot in the training group. **b** Calibration plot in the validation group. The dashed line represents the original performance, and the solid dashed line represents the performance during internal validation by bootstrapping (B = 1000 repetitions)

**Fig. 5:**
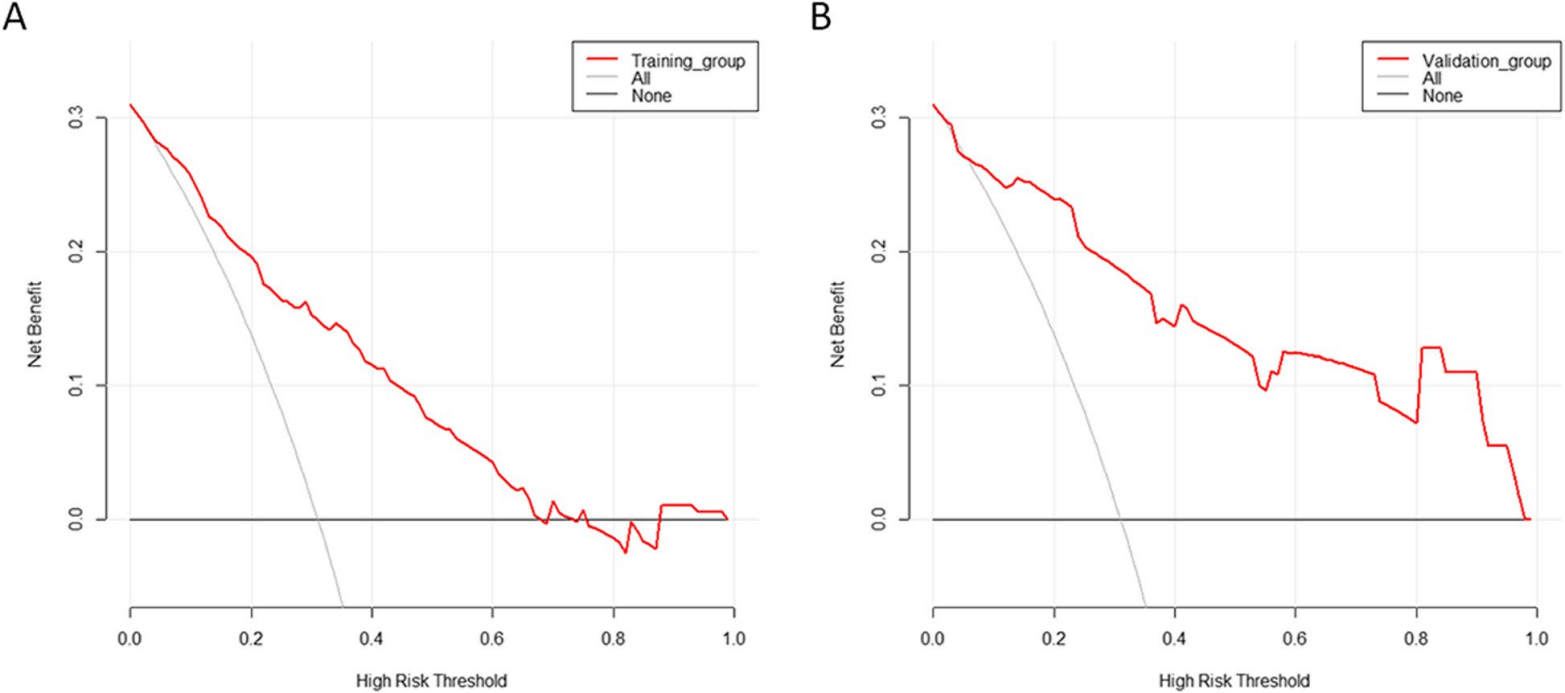
DCA of the nomogram. **a** DCA in the training group. **b** DCA in the validation group. Red-solid line: The patient does not apply the nomogram and the net benefit is zero; Grey-solid line: All patients are treated by the nomogram. The area enclosed by the three lines presents the clinical utility of the nomogram. DCA: decision curve analysis

ROC curve analysis showed that the AUC was 0.814 (95% CI 0.751–0.877), the specificity was 76.8%, and the sensitivity was 74.2% (Fig. 3A). Our model showed high agreement between the actual and predicted probabilities in the training group, with a calibration curve slope close to 1 (Fig. 4A). In addition, the DCA curve demonstrated that our model was clinically useful in the training group (Fig. 5A).

### Validation and evaluation of the prediction model

A total of 66 patients were included in the validation group for external validation of the model, among whom there were 23 (34.8%) with acute hepatic failure, 0 (0%) with subacute hepatic failure, 12 (18.2%) with acute-on-chronic hepatic failure, 13 (19.7%) with chronic hepatic failure and 18 (27.3%) with hepatic dysfunction that did not meet the diagnostic criteria for hepatic failure. There were 49 (74.2%) patients with AKI, 5 (7.6%) patients with chronic kidney disease and 12 (18.2%) patients with other indications for CRRT. As presented in Table 4, there were no significant differences between the two groups, except that the PCO2 was higher in the training group than in the validation group.

**Table 4:** Comparison of characteristics between the training group and validation group

Characteristics	training group (n = 187)	validation group(*n* = 66)	Z/χ2	*P*-value
Age (years)	56(46, 66)	60.5(47.5,71)	−1.591	0.112
Male【n(%)】	128(68.4)	45(68.2)	0.002	0.968
Hepatic dysfunction classification 【n(%)】			2.447	0.654
acute hepatic failure	52(27.8)	23(34.8)		
subacute hepatic failure	3(1.6)	0(0)		
acute-on-chronic hepatic failure	37(19.8)	12(18.2)		
chronic hepatic failure	34(18.2)	13(19.7)		
hepatic dysfunction	61(32.6)	18(27.3)		
Indications for CRRT 【n(%)】			1.424	0.491
CKD	11(5.9)	5(7.6)		
AKI	129(69)	49(74.2)		
Others	47(25.1)	12(18.2)		
BMI (kg/m^2)	22.7(20.3,25.60)	23.6(20.76,27)	−1.324	0.186
Weight (kg)	62.75(55,70)	63(55, 73)	−0.655	0.513
Map (mmHg)	81(74,89)	82(75.5,91)	−1.059	0.290
Norepinephrine (ug/kg/min)	0.02(0, 0.16)	0.01(0,0.11)	−0.328	0.743
Alb (g/L)	33.1(29.3,36.6)	32.65(29.68,36.78)	−0.259	0.795
AST (U/L)	127(53, 438)	181.5(72,478)	−1.068	0.285
ALT (U/L)	102(33, 387)	127(39,479.5)	−0.751	0.452
LDH (U/L)	399(284,758)	506(288,906.25)	−1.139	0.255
Tbil (umol/L)	130(57,257)	113.4(48.88,249.43)	−1.033	0.302
CRP (mg/L)	53.26(20.15,101.55)	57.35(20.98,106.15)	−0.241	0.810
WBC(*10^9/L)	9.65(6.5,15.24)	9.3(5.9,12.2)	−0.732	0.464
PLT(*10^9/L)	46(29,106)	59(32,90)	−0.743	0.458
Hb(g/L)	73(62,93)	78(64,94)	−0.867	0.386
PT(s)	18.1(14.6,24.3)	17.4(15.13,22.65)	−0.170	0.865
INR	1.64(1.28,2.22)	1.52(1.28,1.91)	−1.017	0.309
APTT(s)	44.1(33.2,60.1)	40.75(33.23,55.3)	−0.967	0.334
serum potassium (mmol/L)	4.32(4,4.67)	4.26(3.93,4.76)	−0.766	0.438
tCa (mmol/L)	2.25(2.17,2.25)	2.23(2.15, 2.30)	−1.423	0.155
Scr (umol/L)	168(87,255)	153(82.75, 287.75)	−0.217	0.828
BUN (mmol/L)	14.6(7.9,14.6)	13.8(8.10,26.45)	−0.484	0.629
pH	7.43(7.38,7.47)	7.42(7.36,7.47)	−0.776	0.438
PO2 (mmHg)	106(89,132)	103(82.6123.3)	−1.445	0.148
PCO2 (mmHg)	34.8(31.6,39.2)	33.1(28.93,38.05)	−2.009	0.045
Bicarbonate concentration (mmol/L)	23.16 ± 3.8	22.16 ± 4.20	1.784	0.076
BE (mmol/L)	−1.15 ± 4.13	−2.03 ± 4.74	1.422	0.156
Lactate (mmol/L)	1.9(1.3,3.1)	1.8(1.28,2.58)	−0.569	0.570
iCa (mmol/L)	1.12(1.04,1.18)	1.1(1.03,1.16)	−0.668	0.504
Dialysate flow rate (ml/h)	1000(1000,2000)	1000(1000,2000)	−1.261	0.207
citrate concentration (mmol/L)	3 (2,3)	3(2,3)	−1.286	0.198

*CRR* continuous renal replacement therapy, *AKI* acute kidney injury, *CK* chronic kidney disease, *BMI* body mass index, *MAP* mean arterial pressure, *Alb* albumin, *ALT* alanine aminotransferase, *AST* aspartate aminotransferase, *Tbil* total bilirubin, *LDH* lactate dehydrogenase, *WBC* white blood cell count, *Hb* hemoglobin, *PLT* platelet count, *CRP* C-reactive protein, *APTT* activated partial thromboplastin time, *PT* prothrombin time, *INR* international normalized ratio, *Scr* serum creatinine, *BUN* blood urea nitrogen*, tCa* total calcium concentration, *PO2* partial pressure of oxygen: *PCO2* partial pressure of carbon dioxide, *BE* Base excess, *iCa* ionized calcium concentration

ROC curve analysis of the prediction model in the validation group showed that the AUC was 0.819 (95% CI 0.699–0.938), the specificity was 61.2%, and the sensitivity was 94.1% (Fig. 3B). The model also represented acceptable agreement between the actual and predicted probabilities (Fig. 4B), and the DCA curve demonstrated that our model was clinically useful in different settings (Fig. 5B).

## Discussion

The risk of citrate accumulation, which is a feared complication of citrate anticoagulation, increases in patients with hepatic insufficiency due to the slowed metabolism of citrate. The adverse effects of citrate accumulation can be reduced by frequently monitoring the concentration of calcium and blood gases, but difficulties remain for clinicians in developing anticoagulation protocols. Therefore, constructing a model for predicting the risk of citrate accumulation would be useful for screening high-risk patients before the use of citrate so that the CRRT mode, parameters, and citrate rate can be adjusted appropriately before the start of CRRT, reducing the risk of citrate accumulation.

In our study, citrate accumulation episodes occurred in 33.2% of patients with hepatic insufficiency treated with RCA-CRRT, which was similar to the previously reported 33% incidence of citrate accumulation in patients with hepatic failure and significantly higher than the 12% in normal patients [23].

The causes of citrate accumulation are multifaceted when considering that the probability of citrate accumulation varies among patients with similar severities of liver injury and that even some patients with mild liver injury experience accumulation. In our study, six independent risk factors, including sex, INR, norepinephrine dosage, PO2, peripheral blood citrate concentration and dialysate flow rate, were identified for the development of modeling predicting citrate accumulation in patients with hepatic insufficiency undergoing RCA-CRRT. In addition, the INR and peripheral blood citrate concentration were the main factors influencing the occurrence of citrate accumulation. In contrast to other studies in which lactate was shown to be a risk factor for citrate accumulation [21], our present study did not find that it was a significant predictor, which may be related to the fact that lactate kinetics rather than an initially elevated lactate concentration should be focused on when assessing the risk of citrate accumulation, as Khadzhynov D et al. suggested [24].

Our study also found that the peripheral blood citrate concentration and dialysate flow rate were independent risk factors for citrate accumulation. We used a fixed concentration in the citrate anticoagulation regimen and a fixed blood flow rate but selected a lower dose of citrate and increased the dialysate flow rate for patients with severe hepatic insufficiency to reduce the occurrence of citrate accumulation. As Yessayan, L et al. indicated in 2021 [25], a fixed citrate-to-blood flow ratio could reduce the risk of clinically significant hypocalcemia. Several studies have suggested that reducing the initial citrate concentration in patients with liver disease is beneficial and that citrate dosing regimens should be reduced by at least twofold to a citrate concentration of 1.5 mmol/L in patients with liver failure to avoid citrate accumulation and toxicity [7]. A lower initial citrate dose (peripheral blood citrate concentration of 2.5 mmol/L) for RCA regimens in Asians with smaller bodies has also been recommended, with fewer citrate-related complications and no loss of efficacy [26]. We also found that the median use time of the filter and line was at least 48 hours with low doses of citrate but up to 60 hours when the peripheral blood citrate concentration was 1.5 mmol/L, indicating that reduced doses of citrate can still achieve good results, which may be related to coagulation dysfunction in patients with hepatic insufficiency.

We also found a relationship between the INR and the risk of developing citrate accumulation, which is consistent with the results of previous studies [18, 27]. In our study, female patients were more likely to develop citrate accumulation, possible because women have less muscle, which affects citrate metabolism [28, 29]. Similar to previous studies, a norepinephrine dosage ≥0.1 μg/kg/min, which represents hemodynamic instability and inadequate tissue perfusion [30], was an independent risk factor for citrate accumulation in our study. Oxygen is a key factor in the tricarboxylic acid cycle, and a state of hypoxia can lead to a decrease in citrate metabolism when the efficiency of the tricarboxylic acid cycle decreases, which ultimately causes citrate accumulation [31]. The participants in this study were critically ill patients admitted to the ICU with advanced respiratory support, the oxygen pressure of which was maintained at relatively high levels. We found that PO2 < 80 mmHg was also a risk factor for citrate accumulation.

Few studies have provided simple and convenient prediction models for citrate accumulation, and most recent studies have been focused on the analysis of safety and risk factors for citrate accumulation in hepatic failure patients treated with citrate anticoagulation. X. Xin et al. constructed a model for predicting the risk of accumulation in liver transplant patients receiving citrate anticoagulation [30], the results of which might be biased and lack external validation, making the model less generalized, especially because only 32 patients were included. We developed a new prediction model based on variables that can be obtained quickly in routine clinical examinations and the outcome of which can be easily calculated at the bedside through electronic medical records, which would significantly improve the application of the prediction model in clinical practice. Different from previous models, the constructed prediction model incorporates two variables, the peripheral blood citrate concentration and dialysate flow rate, which could be used as intervening variables compared to fixed variables such as sex. After initial assessment of the patient’s condition, for patients at high risk of citrate accumulation, clinicians can adjust these two variables to view the probability of citrate accumulation after adjustment of CRRT parameters and thereby receive clinical guidance on whether to perform RCA-CRRT or reduce the citrate concentration. The prediction model in this study is more flexible than previous models and provides a basis for clinicians to develop individualized anticoagulation protocols. A nomogram was constructed in our study to analyze influencing factors visually [32, 33]; the underlying model showed good discrimination, calibration and clinical utility. Thus, this tool could help clinicians in assessing the risk of citrate accumulation in patients with hepatic insufficiency undergoing RCA-CRRT anytime, anywhere, easily and quickly and offer the capability to screen patients with a high risk of citrate accumulation for early prevention and intervention.

There are some limitations to this study. First, it was a single-center retrospective study with a relatively small number of cases, leading to selection bias to some extent. Second, while the study was externally validated, the data came from the same center, so whether this nomogram could be widely applied in clinical practice requires further multicenter studies of larger samples.

## Data Availability

The raw data supporting the conclusions of this article will be made available by the first author: Quxia Hong (Email: 809701453@qq.com).

## References

[1] Zarbock A (2020). Effect of regional citrate anticoagulation vs systemic heparin anticoagulation during continuous kidney replacement therapy on Dialysis filter life span and mortality among critically ill patients with acute kidney injury: a randomized clinical trial. Jama..

[2] Bai M (2015). Citrate versus heparin anticoagulation for continuous renal replacement therapy: an updated meta-analysis of RCTs. Intensive Care Med..

[3] Khwaja A (2012). KDIGO clinical practice guidelines for acute kidney injury. Nephron Clin Pract..

[4] Chappell JB (1964). The oxidation of citrate, isocitrate and cis-aconitate by isolated mitochondria. Biochem J..

[5] Honore PM (2019). Inducible metabolic pathway for citrate metabolism in case of major liver dysfunction: fact or fiction?. Crit Care..

[6] Zheng Y (2013). Citrate pharmacokinetics in critically ill patients with acute kidney injury. PLoS One..

[7] Thanapongsatorn P (2022). Citrate pharmacokinetics in critically ill liver failure patients receiving CRRT. Sci Rep..

[8] Apsner R (1997). Impairment of citrate metabolism in acute hepatic failure. Wien Klin Wochenschr..

[9] Morgera S (2004). Metabolic complications during regional citrate anticoagulation in continuous venovenous hemodialysis: single-center experience. Nephron Clin Pract..

[10] Link A, et al. Total-to-ionized calcium ratio predicts mortality in continuous renal replacement therapy with citrate anticoagulation in critically ill patients. Crit Care. 2012; 10.1186/cc11363. 10.1186/cc11363 PMC3580644 22643456

[11] Slowinski T (2015). Safety and efficacy of regional citrate anticoagulation in continuous venovenous hemodialysis in the presence of liver failure: the liver citrate anticoagulation threshold (L-CAT) observational study. Crit Care..

[12] Zhang W, et al. Safety and efficacy of regional citrate anticoagulation for continuous renal replacement therapy in liver failure patients: a systematic review and meta-analysis. Crit Care. 2019; 10.1186/s13054-019-2317-9. 10.1186/s13054-019-2317-9 PMC6345001 30678706

[13] Boer W (2020). Determinants of Total/ionized calcium in patients undergoing citrate CVVH: a retrospective observational study. J Crit Care..

[14] Faybik P, Hetz H (2006). Plasma disappearance rate of Indocyanine green in liver dysfunction. Transplant Proc..

[15] Kramer L (2007). Incidence and prognosis of early hepatic dysfunction in critically ill patients—a prospective multicenter study. Crit Care Med..

[16] Patel ST (2022). Hepatic dysfunction in medical intensive CAREUNIT patients predicts poor outcome. Arq Gastroenterol..

[17] Hetzel GR (2006). Citrate plasma levels in patients under regional anticoagulation in continuous venovenous hemofiltration. Am J Kidney Dis..

[18] Schultheiß C (2012). Continuous venovenous hemodialysis with regional citrate anticoagulation in patients with liver failure: a prospective observational study. Crit Care..

[19] Clegg A, Young J, Iliffe S, Rikkert MO, Rockwood K (2013). Frailty in elderly people. Lancet..

[20] [Guideline for diagnosis and treatment of liver failure]. Zhonghua Gan Zang Bing Za Zhi 27, 18–26 (2019) 10.3760/cma.j.issn.1007-3418.2019.01.006 10.3760/cma.j.issn.1007-3418.2019.01.006 PMC12770200 30685919

[21] Tan JN (2019). Hyperlactatemia predicts citrate intolerance with regional citrate anticoagulation during continuous renal replacement therapy. J Intensive Care Med..

[22] Lambden S, Laterre PF, Levy MM, Francois B (2019). The SOFA score-development, utility and challenges of accurate assessment in clinical trials. Crit Care..

[23] Meier-Kriesche HU, Gitomer J, Finkel K, DuBose T (2001). Increased total to ionized calcium ratio during continuous venovenous hemodialysis with regional citrate anticoagulation. Crit Care Med..

[24] Khadzhynov D (2017). Hyperlactatemia, lactate kinetics and prediction of citrate accumulation in critically ill patients undergoing continuous renal replacement therapy with regional citrate anticoagulation. Crit Care Med..

[25] Yessayan L (2021). Regional citrate anticoagulation "non-shock" protocol with pre-calculated flow settings for patients with at least 6 L/hour liver citrate clearance. BMC Nephrol..

[26] Poh CB (2020). Regional citrate anticoagulation for continuous renal replacement therapy - a safe and effective low-dose protocol. Nephrology (Carlton)..

[27] Klingele M (2017). Long-term continuous renal replacement therapy and anticoagulation with citrate in critically ill patients with severe liver dysfunction. Crit Care..

[28] Wiik A, et al. Muscle strength, size, and composition following 12 months of gender-affirming treatment in transgender individuals. J Clin Endocrinol Metab. 2020;105 10.1210/clinem/dgz247. 10.1210/clinem/dgz247 31794605

[29] Ma Y (2020). A novel predictive score for citrate accumulation among patients receiving artificial liver support system therapy with regional citrate anticoagulation. Sci Rep..

[30] Xin X (2022). Development of a multivariable prediction model for citrate accumulation in liver transplant patients undergoing continuous renal replacement therapy with regional citrate anticoagulation. Blood Purif..

[31] Fuhrmann DC, Brüne B (2017). Mitochondrial composition and function under the control of hypoxia. Redox Biol..

[32] Zhang Z, Kattan MW (2017). Drawing nomograms with R: applications to categorical outcome and survival data. Ann Transl Med..

[33] Zhang M, Hu ZD (2019). Suggestions for designing studies investigating diagnostic accuracy of biomarkers. Ann Transl Med..

